# Role of GLP-1 in the Hypoglycemic Effects of Wild Bitter Gourd

**DOI:** 10.1155/2013/625892

**Published:** 2013-03-26

**Authors:** Ting-ni Huang, Kan-Ni Lu, Yi-Ping Pai, Chin Hsu, Ching-jang Huang

**Affiliations:** Department of Biochemical Science and Technology, National Taiwan University, Taipei 106, Taiwan

## Abstract

This study aimed to examine the role of GLP-1 in the hypoglycemic activity of wild bitter gourd (*Momordica charantia* L., BG). In vitro, the GLP-1 secretion in STC-1, a murine enteroendocrine cell line, was dose dependently stimulated by water extract (WE), its fractions (WEL, >3 kD and WES, <3 kD), and a bitter compounds-rich fraction of BG. These stimulations were partially inhibited by probenecid, a bitter taste receptor inhibitor, and by U-73122, a phospholipase C**β**2 inhibitor. These results suggested that the stimulation might involve, at least in part, certain bitter taste receptors and/or PLC**β**2-signaling pathway. Two cucurbitane triterpenoids isolated from BG, 19-nor-cucurbita-5(10),6,8,22-(E),24-pentaen-3**β**-ol, and 5**β**,19-epoxycucurbita-6,24-diene-3**β**,23**ξ**-diol (karavilagenine E,) showed relative high efficacy in the stimulation. In vivo, mice fed BG diet showed higher insulinogenic index in an oral glucose tolerance test. A single oral dose of WE or WES pretreatment significantly improved intraperitoneal glucose tolerance. A single oral dose of WES significantly decreased glucose and increased insulin and GLP-1 in serum after 30 min. This acute hypoglycemic effect of WES was abolished by pretreatment with exendin-9, a GLP-1 receptor antagonist. Our data provide evidence that BG stimulates GLP-1 secretion which contributes, at least in part, to the antidiabetic activity of BG through an incretin effect.

## 1. Introduction

In response to food ingestion, enteroendocrine cells in the intestinal mucosa release hormones that can stimulate insulin secretion from endocrine pancreas and thereby reduce blood glucose [1, 2]. This is known as the incretin effect [1, 2] and two such hormones, that is, GLP-1 and GIP, have been identified as the incretins [1, 2]. The insulin secretory response to incretins accounts for at least 50% of the total insulin secreted after oral glucose. Despite that lacking secretion or faster clearance of incretin are not pathogenic factors in diabetes, GLP-1 has become a molecular target for therapeutics of type 2 diabetes mellitus (type 2 DM) since its insulinotropic activity is still active in these patients, but not GIP [1, 2]. Two such strategies have already been in clinical practice to treat type 2 DM, namely, GLP-1 analogs and inhibitors of the enzyme dipeptidylpeptidase IV (DPP IV) that degrades both GLP-1 and GIP [1]. 

The GLP-1 biology has been extensively reviewed [1–4]. This endocrine hormone is produced and secreted in the enteroendocrine L cells and its major physiological roles include: (1) the stimulation of glucose-dependent insulin secretion from pancreatic *β*-cells, (2) stimulation of insulin biosynthesis and insulin sensitivity, (3) enhancement of pancreatic *β*-cell proliferation and protection against apoptosis, (4) inhibition of glucagon secretion and gastric emptying, and (5) inhibition of food intake [1–4].

An alternative approach to target GLP-1 is the identification of GLP-1 secretagogues that have the potential, either alone or in combination with DPP IV inhibitors to enhance circulating levels of bioactive GLP-1 in patients with T2DM [4]. In this respect, the receptors expressed on intestinal L cells that can transduce cell signaling and evoked GLP-1 secretion are especially of interests [5, 6]. For example, GPR40 [7] and GPR120 [8] that bind specific types of fatty acids, GPR 119 [9] that binds oleoylethanolamide (a fatty acid derivative) and lysophosphatidylcholine, and TGR5 [10] that binds bile acids are linked to GLP-1 secretion. Whereas enteroendocrine cells including L cells are sensors for nutrients and food components ingested and mediate physiological responses, GLP-1 secretion is also stimulated by nutrients and dietary components [11]. Some, but not all, receptors responsible for the stimulation by nutrients have been characterized [11]. 

Interestingly, sweet taste receptors are also expressed in the L cells and considered targets for developing GLP-1 based therapeutics [12, 13]. The sweet receptor, T1R2+T1R3, and its signaling pathway in L cells have been shown to regulate oral glucose-stimulated GLP-1 secretion [14]. The applications of this finding in clinical trials have not been successful to date [15–17]. There is also evidence showing that the T2R bitter taste receptors are expressed in the enteroendocrine cells in gut and cell lines [18–20]. Dotson et al. [21] found that a functionally compromised TAS2R bitter taste receptor negatively impacts glucose homeostasis. They also showed that the stimulation of the NCI-H716 cells with ofloxacin (a TAS2R ligand) elicited a dose-dependent secretion of GLP-1 from this human enteroendocrine cell line.

Bitter gourd (*Momordica charantia*, BG) is a common tropical vegetable that has also been used to manage diabetes in oriental traditional medicine. The antidiabetic effects of BG have been extensively reviewed [22–24]. The multiple mechanisms underlied [25] and the active components have also been reviewed [26]. The possible mechanisms reported include inhibiting intestinal *α*-glucosidase [27] and glucose transport [28], protecting islet *β*-cells [29], enhancing insulin secretion [30], increasing hepatic glucose disposal and decreasing gluconeogenesis [31–33], activating AMPK [34, 35], and improving insulin resistance [36, 37]. We previously reported that BG extracts can activate and upregulate the expressions of peroxisome proliferator-activated receptors (PPARs) and identified 9c, 11t, 13t-conjugated linolenic acid as one of the active components [38, 39]. In a preliminary clinical study with 42 metabolic syndrome subjects, the incidence of metabolic syndrome and waist circumference was significantly decreased after a supplementation of 4.8 g/d of lyophilized wild bitter gourd powder (BGP) for 3 months [40].

One of the known functions of GLP-1 is the enhancement of pancreatic *β*-cell proliferation and protection against apoptosis [3]. The administration of BG juice to diabetic rats for 9 weeks significantly increased the number of *β* cells [29]. On the other hand, the bitter tasting compounds of BG might activate bitter taste receptors in the L cells and elicit more GLP-1 secretion. To test the hypothesis that BG might stimulate GLP-1 secretion and exert an incretin effect to regulate glucose homeostasis, we measured GLP-1 secretion in STC-1 cells and mice treated with BG extracts. Furthermore, we found that the acute hypoglycemic effect of BG extract in mice was abolished by *Exendin-9*, a GLP-1 antagonist. 

## 2. Materials and Methods

### 2.1. Materials

Fresh Hualien No.4 wild bitter gourds (BG) were provided by Hualien Agricultural Experiment Station. Whole fruits (with seeds) were washed, sliced, frozen, and freeze dried. The lyophilized BG slices were grounded to obtain lyophilized BG powder (BGP). Oleanolic acid, palmitic acid (PA), probenecid, and U73122 were from Sigma Co. 9c, 11t, 13t-conjugated linolenic acid (CLN), oleic acid (OA), linoleic acid (LA), and linolenic acid (LN) were from Cayman Co. Conjugated linoleic acid (CLA, a 50%/50% mixture of c9,ct11- and t10, c12- isoforms) and linolenic acid (LN) were from Calrinol Co. (Holland). Pure triterpenoids compounds isolated from BG, including compound 1, cucurbita-6,22(E),24-trien-3*β*-ol-19,5*β*-olide; compound 2, 5*β*,19-epoxycucurbita-6,22(E),24-triene-3*β*,19-diol; compound 3, 3*β*-hydroxycucurbita-5(10),6,22(E),24-tetraen-19-al; compound 4, 19-dimethoxycucurbita-5(10),6,22(E),24-tetraen-3*β*-ol; compound 5, 19-nor-cucurbita-5(10),6,8,22-(E),24-pentaen-3*β*-ol; compound 6, 5*β*,19-epoxycucurbita-6,24-diene-3*β*,23*ξ*-diol (karavilagenine E), were from our previous study [41]. 

### 2.2. Preparation of Water Extract (WE), Ethyl Acetate Extract (EAE), and Ethanol Extract (EE)

Lyophilized BGP was, respectively, extracted with dH_2_O or ethyl acetate (1 : 20, w/v) with stirring at room temperature overnight. The water extract (WE) was obtained by centrifugation (3000 ×g, 10 min), filtration and freeze-drying. The ethyl acetate extract (EAE) was obtained by filtration, and evaporation to remove the solvent. The residue from EA extraction was also evaporated to remove residual EA and continued extracted with ethanol (1 : 20, w/v) with stirring at room temperature overnight. The ethanol extract was also obtained by filtration and evaporation to remove the solvent. The extraction rates of WE, EAE, and EE were 27%, 4.8%, and 16.4%, respectively.

### 2.3. Fractionation of WE to Large (>3 kD, WEL) and Small (<3 kD, WES) Molecule Fractions

Lyophilized WE powder was dissolved in dH_2_O. The solution was separated by ultrafiltration with a 3 kD molecular weight cutoff filter devices (Millipore). After centrifugation, the residue that remained in the filter device sample reservoir was collected to obtain WEL, the fraction that contained >3 kD molecules. The ultrafiltrate in the centrifuge tube was collected to obtain WES, the fraction that contains <3 kD molecules. WEL and WES were also lyophilized and the yields were 14% and 86%, respectively. 

### 2.4. Preparation of Insulin-Like Peptide-Rich Fraction (Pf) from WE

An insulin-like peptide (the so-called “plant-insulin”) has been isolated from BG. This peptide is structurally and pharmacologically similar to bovine insulin [42]. The insulin-like peptide-rich fraction was prepared according to the method of Khanna et al. with slight modification [42]. Lyophilized WE powder was stirred in acidic-ethanol (0.05 M H_2_SO_4_, 60% ethanol, 1 : 20, w/v) at 4°C overnight. The mixture was filtered. Ammonium hydroxide (28%) was added to the filtrate to adjust the pH to 3. The filtrate was then added with acetone (1 : 4, v/v) to form precipitate at 4°C overnight. The precipitate was dialyzed in a dialysis membrane with 3 kD molecular weight cutoff to remove salt and impurities. The nondialyzable fraction was collected and freeze dried to obtain Pf.

### 2.5. Extraction of Crude Bitter Taste Extract (BGP-bi) from BGP

The bitter taste of BG fruits has been attributed to momordicoside K and L which are cucurbitane-type triterpenoid glycoside [43]. A fraction rich in the two momordicosides was prepared according to the method of Okabe et al. [43] with slight modifications. Lyophilized BGP was stirred in dH_2_O and methanol (1 : 9 : 20, w/v/v) at room temperature overnight. After the mixture was filtered, the filtrate was concentrated to 1/10 of its original volume in a rotary evaporator. The concentrated filtrate was partitioned with CHCl_3_ (1 : 1.5, v/v) in a separating funnel overnight. The chloroform extract obtained was concentrated to dryness in a rotary evaporator to obtain BGP-bi, the bitter taste triterpenoid glycoside rich fraction.

### 2.6. GLP-1 Secretion of STC-1 Cells

 STC-1 cells, a murine enteroendocrine cell line, were provided by Dr D Hanahan at University of California, San Francisco. Cells were grown in 10% fetal bovine serum (FBS; Gibco) DMEM (high glucose) at 37°C and 5%CO_2_ till near confluence. The procedure for measuring GLP-1 secretion of STC-1 cells was modified from the report of Eiki et al. [44]. Cells were trypsinized and seeded in 48 well plates (6 × 10^4^ cells per well) with DMEM (4.5 g/L glucose, Gibco) supplemented with 10% (v/v) FBS for 2 days. Culture media were then replaced by the low glucose DMEM (1 g/L glucose) containing 10% FBS for glucose starvation and incubation continued for additional 3 hrs. Cells were then treated with test samples (BG extracts or compounds) or vehicle at 37°C and 5% CO_2_ for 1 hr. Test samples dissolved in dH_2_O or absolute ethanol were diluted in DMEM (1 g/L glucose) that contained 0.2% (w/v) BSA (Sigma). When samples were dissolved in absolute ethanol, the parallel vehicles also contained ethanol in the amount that is equivalent to the highest concentrations of test samples. The concentration range of each sample that did not significantly affect cell viability was used in treating cells. After 1 hr of incubation, medium was collected and centrifuged at 500 g and 4°C for 5 min. GLP-1 content in the supernatant was determined by an ELISA kit (Millipore). Numbers of cells in the plate were measured by the MTT (Sigma) assay. Results were presented as GLP-1 secretion per cell. In some experiments, Probenecid (Sigma), an inhibitor of bitter taste receptors [45], or U73122 (Sigma), a PLC*β*2 inhibitor, was added to examine the role of bitter taste receptors in the GLP-1 production. 

### 2.7. Animal Studies

The animal study was approved by the Institutional Animal Care and Use Committee, National Taiwan University (No. NTU-98-EL-20). Eight-week old male C57BL/6J mice were purchased from the National Laboratory Animal Center (Taipei, Taiwan). Mice were individually housed in stainless steel wire cages in an animal room with a 12-h light, 12-h dark cycle and constant temperature (22 ± 2°C). For acclimatization, mice were fed a non-purified diet (Rodent Chow, PMI Nutrition International, Brentwood, MO USA). 

For acute effect (including the exendin-9) experiments described below, mice were a high fat diet (30%, w/w, 29% butter) [46] until their body weight reach 26 or 30 g. Mice had free access to water and diet unless specified below.

### 2.8. Oral Glucose Tolerance and Insulinogenicindex in Mice Fed the BGP Diet for 5 Weeks

Mice were ad-libitum fed either a basal diet or a BGP diet for 5 weeks. The basal diet was a modified AIN-93G [47] diet in which carbohydrate sources were provided by 50% sucrose and 12.95% cornstarch. The BGP diet was formulated by incorporating 5% (w/w) of BGP into the basal diet with adjustment of the composition of casein, corn starch, soybean oil and cellulose based on the proximate composition of BGP, that is, crude protein 4.5%, carbohydrate 54.6%, crude fat 2.7% and dietary fiber 38.2%. As a result, both diets had equal amount of fiber (5% w/w) and provided similar calories from carbohydrate (64%), protein (20%) and fat (16%). After 5 weeks feeding, mice were subjected to an oral glucose tolerance test (OGTT). Mice were feed-deprived overnight and orally fed a glucose solution at the dose of 2 g/kg body weight. Blood samples were collected from retro-orbital at 0, 15, 30, 60 and 90 min. Collected blood samples were centrifuged at 12000 ×g, 4°C for 20 min and serum isolated for the analysis of glucose on the same day as described below. The insulinogenic index was calculated as the ratio of change in serum insulin concentration (15 min–0 min)/change in serum glucose concentration (15 min–0 min).

### 2.9. Acute Effects of WE, WEL and WES on Serum Glucose in an Intraperitoneal Glucose Tolerance Test (ipGTT)

The high fat diet fed mice that weighed ~30 g were fasted for 6 h and orally administered with samples (WE, WES or WEL) or vehicle (*n* = 3~4/group). Doses of samples were: 2100 mg/kgBW of WE, 1800 mg/kgBW of WES, or 300 mg/kgBW of WEL. As these preparations contained glucose (27~86 mg/g), the respective vehicle contained an equal amount of glucose as in the extract/fractions. Thirty min after the administration, mice were intra-peritoneally injected a glucose solution at the dose of 1 g/kg body weight. Blood samples were collected from retro-orbital at 0, 15, 30, 60 and 90 min. The collected blood samples were centrifuged at 12000 ×g, 4°C for 20 min and serum isolated for the analysis of glucose on the same day as described below. 

### 2.10. Acute Effects of WES on Blood Glucose, Insulin and GLP-1 Levels

The high fat diet fed mice that weighed ~26 g were fasted for 6 hrs. At the beginning of the experiment, blood samples were taken from retro-orbital at time 0. Mice were then gavaged with WES at a dose of 5000 mg/kg or vehicle that contained an equal amount of glucose as in WES (430 mg glucose/kg BW in distilled H_2_O). Second blood samples were taken 30 min later. For the determination of GLP-1, blood samples were collected into eppendorf tubes containing EDTA and DPP4 inhibitors (Millipore) to avoid the degradation of GLP-1. Blood samples were centrifuged at 4°C to obtain plasma for the analysis of glucose, insulin and GLP-1.

### 2.11. Effects of Exendin-9 on the Acute Hypoglycemic Effect of WES

The high fat diet fed mice that weighed ~26 g were fasted for 6 hrs. At the beginning of the experiment, blood samples were taken from retro-orbital at time 0. Exendin-9 (a GLP-1 antagonist, American peptide) or vehicle (5 *μ*L PBS/g Body weight), was administered at a dose of 0.12 mg/kg BW via intra-peritoneal injection. Five minutes later, mice were then gavaged with WES at a dose of 3000 mg/kg or vehicle that contained an equal amount of glucose as in WES (258 mg glucose/kg BW in distilled H_2_O). Blood samples were taken from retro-orbital at 30, 60, 120 and 180 min after the gavage of WES or vehicle. Blood samples were centrifuged at 4°C to obtain plasma for the analysis of glucose. 

### 2.12. Biochemical Determination

 Glucose concentrations in serum, plasma, WE and WES were measured by enzymatic methods using commercial kits for glucose (Randox Laboratory). Plasma insulin was determined by rat/mouse insulin ELISA kit (Millipore). Cultured medium and plasma GLP-1 levels were determined using active GLP-1 [7–36] ELISA kit (Millipore). 

### 2.13. Statistical Analysis

All values are presented as means ± SD. Data were analyzed by Student's *t* test and RCBD to adjust for different batches of experiments using Statistical Analysis System Software (SAS 9.0) or Microsoft Office 2007/Excel 2007. Some data of Table 1 were analyzed by paired *t* test to compare the differences before and after treatment in a same mouse using Graphpad prism 5. *P* values < 0.05 were considered statistically significant. 

## 3. Results

### 3.1. Insulinogenic Effect of BGP

**Figure 1:**
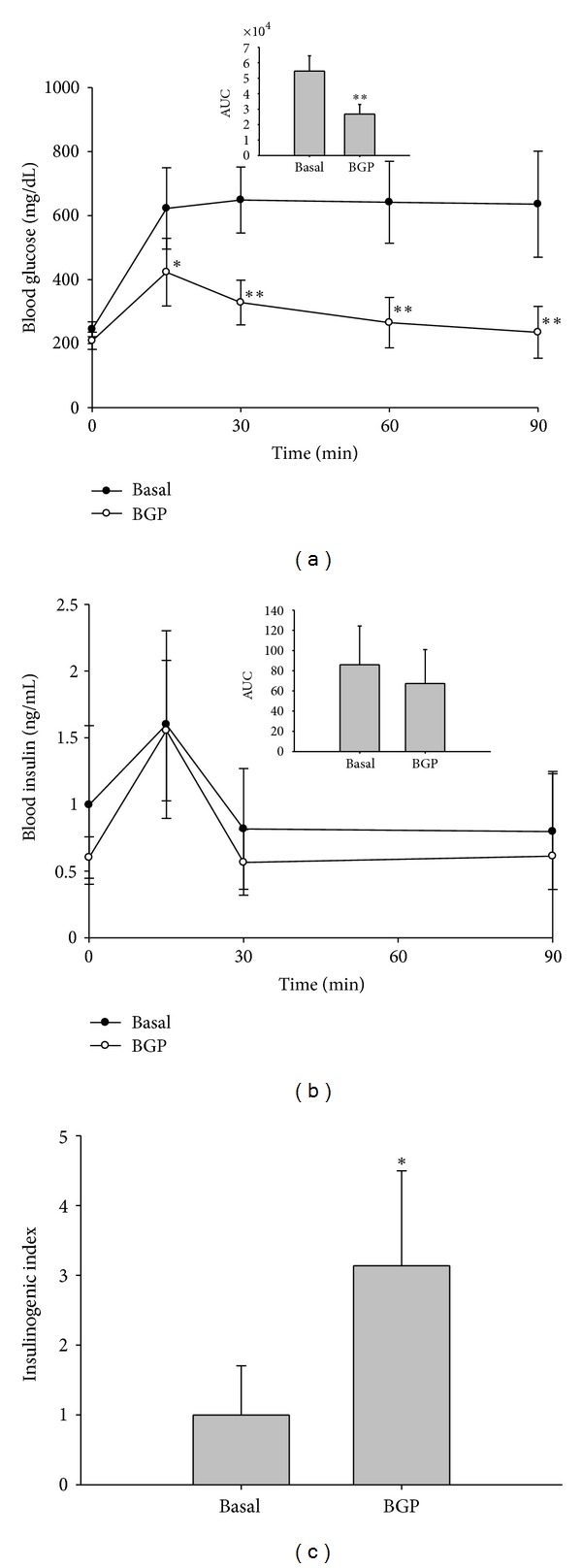
Oral glucose tolerance and insulinogenic index of mice fed the BGP diet for 5 weeks. (a) Serum glucose and area under curve (AUC), (b) serum insulin and area under curve (AUC), and (c) insulinogenic index in an oral glucose tolerance test (OGTT). C57BL/6J male mice were fed a basal or a 5% BGP diet for 5 weeks. For OGTT, mice were feed deprived overnight and orally fed a glucose solution at the dose of 2 g/kg body weight. Insulinogenic index was calculated as the ratio of Δ serum insulin (15 min–0 min)/Δ serum glucose (15 min–0 min), and that of the basal group was taken as 1. Data are mean ± SD. *P < 0.05 and **P < 0.01 denote significant difference compared to the basal group analyzed by Student's *t* test.

As GLP-1 is known to increase insulin secretion, we first examined whether BGP has an insulinogenic effect. Mice were fed a 5% BGP containing diet for 5 weeks and then subjected to an oral glucose tolerance test. As shown in Figure 1(a), the BGP group of mice showed a significantly lower serum glucose level at all time points after the glucose challenge (*P* < 0.05). The remarkably reduced area under curve values, again, indicated a better glucose tolerance in this group. Despite that the serum insulin levels were not significantly different at each time points (Figure 1(b)), the insulinogenic index, calculated as the ratio of changes in plasma insulin 15 min after oral glucose administration and changes in plasma glucose 15 min after oral glucose administration, was significantly higher in the BGP group, compared to that of the basal group (Figure 1(c)) (Basal versus BGP: 0.98 ± 0.69 versus 3.07 ± 1.33, *P* < 0.05). 

### 3.2. WE and Its Fractions Stimulates GLP-1 Secretion in STC-1 Cells

**Figure 2:**
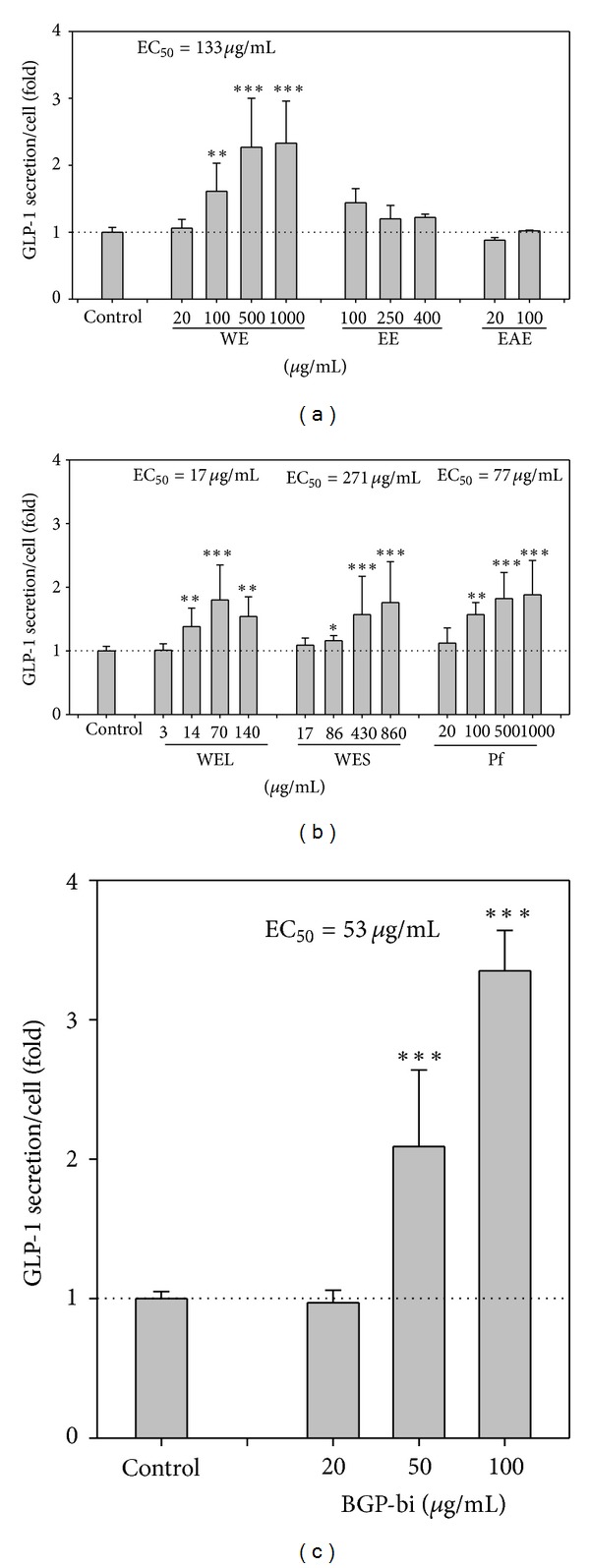
Effects of various BG extracts on GLP-1 secretion in STC-1. After a 3 hr starvation in 10% FBS DMEM (low glucose), cells were treated with 0.2 % BSA DMEM (low glucose) containing (a) WE: water extract, EE: ethanol extract, or EAE: ethyl acetate extract; (b) WEL: large molecular weight fraction, >3 kD, of WE, WES: small molecular weight fraction, <3 kD, of WE, or Pf: P fraction, insulin-like peptide-rich fraction; (c) BGP-bi: crude bitter taste compounds extract for 1 hr. All data were calculated as GLP-1 secretion per cell and the secretion of vehicle-treated cells was taken as 1. The basal GLP-1 secretion of the vehicle-treated cells was 40~120 pM/well. Data are mean ± SD of 1~3 batches of experiments, with *n* = 1~3 for each treatment. *P < 0.05, **P < 0.01, and ***P < 0.001 denote significant difference compared to vehicle-treated cells analyzed by Student's *t* test with RCBD to adjust for the differences between separate batches of experiments.

 To examine whether BG stimulates GLP-1 secretion in L cell, we measured the GLP-1 secretion of STC-1 cells, a murine enteroendocrine cell line, treated with various BGP extracts. As shown in Figure 2(a), WE, the water extract of BGP, dose-dependently increased GLP-1 secretion. At the concentration of 1000 *μ*g/mL, a maximal activation was reached which was 2.33-fold that of vehicle-treated cells. However, neither EE nor EAE had significant effects. Therefore, WE was further separated into WEL (>3 kD) and WES (<3 kD) fractions using a filter with 3 kD molecular weight cutoff. The so called “insulin-like peptide” rich fraction (Pf) was also extracted from WE. All these fractions from WE induced GLP-1 secretion in a dose-dependent manner. The maximal stimulation by WEL, WES and Pf was 1.80 (at 70 *μ*g/mL), 1.76 (at 860 *μ*g/mL) and 1.88 (at 1000 *μ*g/mL)-fold that of the vehicle-treated cells, respectively (Figure 2(b)). Besides, BGP-bi also dose-dependently induced GLP-1 secretion to a maximal stimulation of 3.35-fold that of the vehicle-treated cells, at the concentration of 100 *μ*g/mL (Figure 2(c)). Based on these data, crude extract of BG can stimulate GLP-1 secretion in this enteroendocrine cell line.

**Figure 3:**
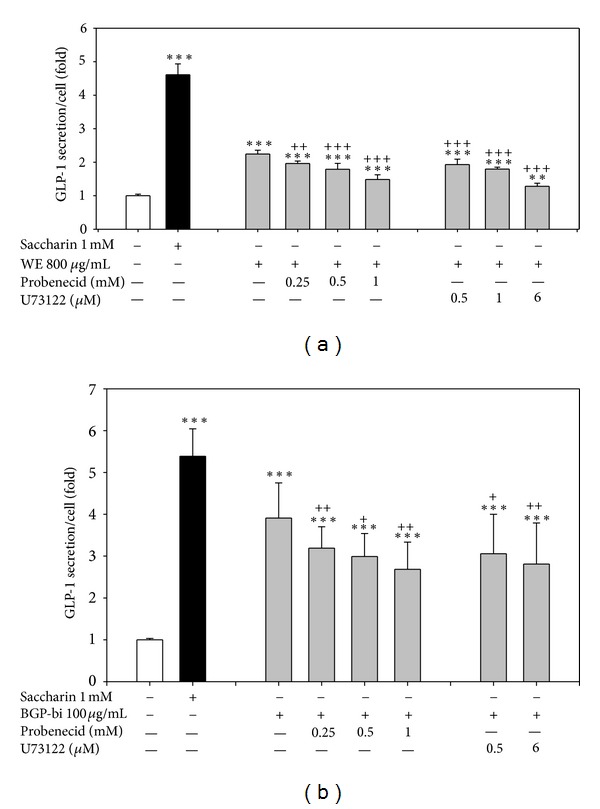
Effects of probenecid or U73122 on the GLP-1 secretion stimulated by WE or BGP-Pi in STC-1. After a 3 hr starvation in 10% FBS DMEM (low glucose), cells were treated with 0.2 % BSA DMEM (low glucose) containing (a) WE (water extract) or (b) BGP-bi in the absence or presence of probenecid or U73122 for 1 hr. All data were calculated as GLP-1 secretion per cell, and the secretion of vehicle-treated cells was taken as 1. Data are mean ± SD of 1~3 batches of experiments, with *n* = 1~3 for each treatment. *P < 0.05, **P < 0.01, and ***P < 0.001 denote significant difference compared to vehicle-treated cells analyzed by Student's *t* test with RCBD to adjust for the differences between separate experiments.

Probenecid can inhibit human TAS2R16 bitter taste receptor [45] and this receptor (TAS2R16) mediates bitter taste in response to *β*-glucopyranosides [48]. The response to two bitter tastants (denatonium and caffeine), in the STC-1 cells can be inhibited by U-73122, a phospholipase C*β*2 inhibitor [49]. To preliminarily test whether bitter taste receptor or phospholipase C*β*2 might be involved in the stimulation of GLP-1 production by WE and BGP-bi, STC-1 cells were treated with WE or BGP-bi in the presence of probenecid or U73122. Both agents partially inhibited GLP-1 production by WE and BGP-bi (*P* < 0.05) (Figure 3). These results suggest that bitter taste receptor and phospholipase C*β*2 signaling pathway might be involved, at least in part, in the GLP-1 production stimulated by WE and BGP-bi. 

### 3.3. GLP-1 Secretion Stimulated by Some Pure Compounds Isolated from BGP

**Figure 4:**
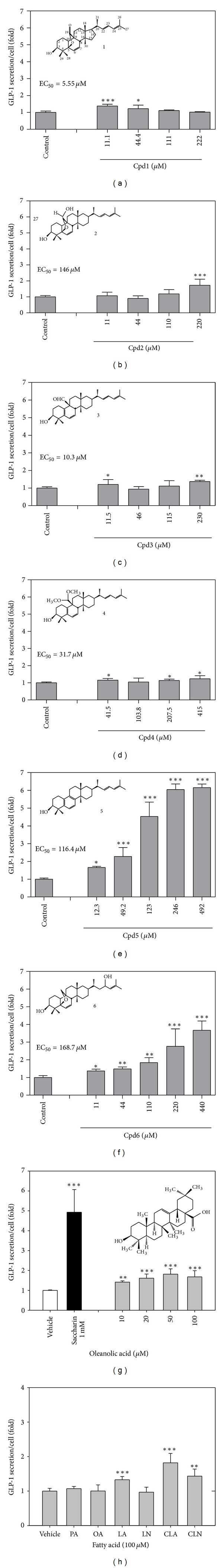
Effects of various BG compounds on GLP-1 secretion in STC-1. After a 3 hr starvation in 10% FBS DMEM (low glucose), cells were treated with 0.2% BSA DMEM (low glucose) containing tested compounds for 1 hr. Tested compounds include (a) compound 1, cucurbita-6,22(E),24-trien-3*β*-ol-19,5*β*-olide; (b) compound 2, 5*β*,19-epoxycucurbita-6,22(E),24-triene-3*β*,19-diol; (c) compound 3, 3*β*-hydroxycucurbita-5(10),6,22(E),24-tetraen-19-al; (d) compound 4, 19-dimethoxycucurbita-5(10),6,22(E),24-tetraen-3*β*-ol; (e) compound 5, 19-nor-cucurbita-5(10),6,8,22-(E),24-pentaen-3*β*-ol; (f) Compound 6, 5*β*,19-epoxycucurbita-6,24-diene-3*β*,23*ξ*-diol (karavilagenine E,); (g) oleanolic acid; (h) conjugated linolenic acid (CLN) and other fatty acids (PA, palmitic acid; OA, oleic acid; LA, linoleic acid; LN, Linoleic acid; CLA, conjugated linoleic acids). All data were calculated as GLP-1 secretion per cell, and the secretion of vehicle-treated cells was taken as 1. Data shown are mean ± SD of 1~3 batches of experiments, with *n* = 1~3 for each treatment. *P < 0.05, **P < 0.01, and ***P < 0.001 denote significant difference compared to vehicle-treated cells analyzed by Student's *t* test with RCBD to adjust for the differences between separate experiments.

We next tested some pure compounds that have been isolated from BGP, including: CLN, oleanolic acid, and 6 cucurbitane-type triterpenoid compounds we previously isolated from BGP [41]. Among these 6 compounds, Compounds 5 and 6 (Figures 4(e) and 4(f)), but not Compound 1–4 (Figures 4(a)–4(d)), significantly and dose-dependently increased GLP-1 secretion in the STC-1 cells. The maximal stimulations reached >6 folds (at 246 *μ*M of Compound 5) and >3 folds (at 440 *μ*M of Compound 6) that of the vehicle-treated cells. In addition, Oleanolic acid and CLN also significantly increased GLP-1 secretion, but to a much less extent (Figures 4(g) and 4(h)). 

### 3.4. Acute Effect of WE, WES and WEL on Intraperitoneal Glucose Tolerance (ipGTT) in Mice

**Figure 5:**
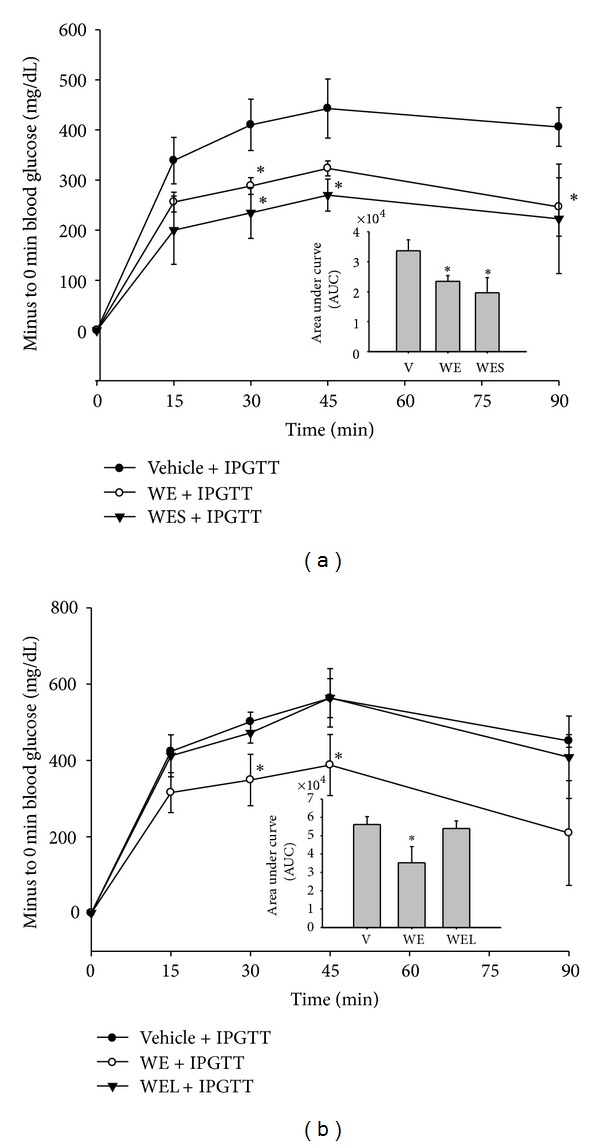
Acute effects of WE, WEL, and WES on serum glucose concentrations in an intraperitoneal glucose tolerance test (ipGTT). High-fat-fed mice were fasted for 6 hr and orally administered with a single dose of WE (2100 mg/kgBW), WES (1800 mg/kgBW) or WEL (300 mg/kgBW) or vehicle containing equivalent amount of glucose as in WE. Thirty minutes later, they were i.p. injected with 1 g/kg BW glucose for ipGTT. Changes in serum glucose were shown in (a) WE, WES versus Vehicle and (b) WE, WEL versus vehicle. Data are mean ± SD. *P < 0.05 and **P < 0.01 denote significant difference compared to vehicle-treated mice analyzed by Student's *t* test.

 The acute effects of WE, WES and WEL on glucose homeostasis control were examined in mice administered with a single dose of these samples and followed by ipGTT. As shown in Figure 5(a), a single dose of WE or WES administered orally significantly improved the disposition of glucose administered intraperitoneally. In contrast, WEL showed no effect (Figure 5(b)).

### 3.5. WES Increases Plasma GLP-1 and Insulin in Mice

 To examine whether WES stimulates GLP-1 secretion in vivo, mice were orally administered a single dose of WES or vehicle containing equal amount of glucose. Serum glucose, insulin and GLP-1 were monitored before and 30 min after the oral administration. Before the oral administration, no significant differences in these blood parameters were observed between the two groups (Table 1). After 30 min, mice orally fed WES, compared to those fed the vehicle, displayed significantly lower glucose, higher insulin and GLP-1 in serum (*P* < 0.05) (Table 1). In addition, these parameters before and 30 min after administration were further compared. It was found that serum glucose significantly decreased and GLP-1 increased 30 min after the administration of WES but not vehicle (*P* < 0.05) (Table 1). On the other hand, no change of plasma DPP4 activity was observed in both groups of mice (data not shown). These data demonstrated that WES also stimulated GLP-1 secretion in vivo.

### 3.6. Exendin-9 Abolished the Acute Hypoglycemic Effect of WES

**Figure 6:**
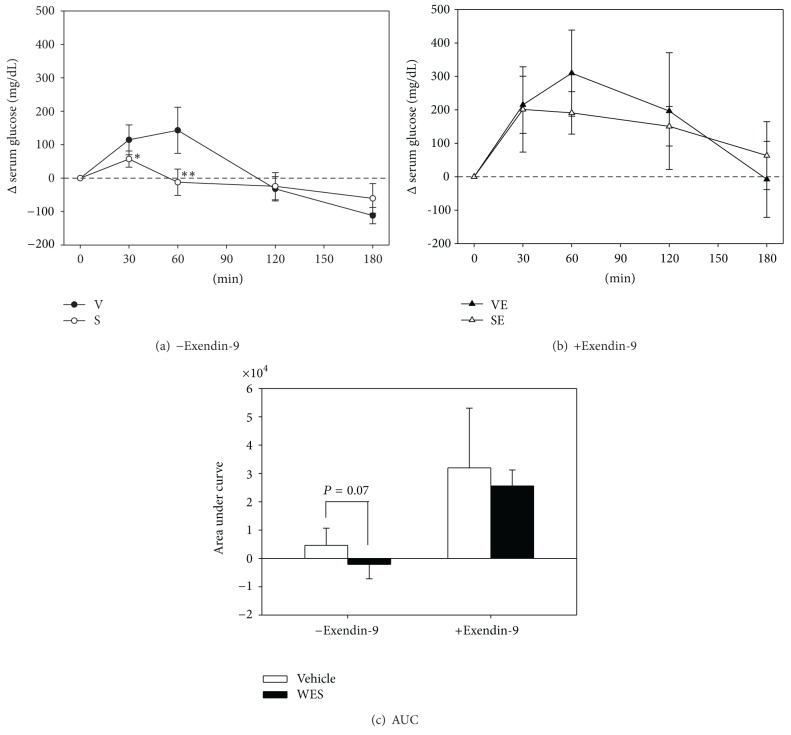
Effects of exendin-9 on the acute hypoglycemic effect of WES. Changes in plasma glucose concentration after a single oral dose of WES to high-fat-diet fed mice without (a) or with (b) a prior administration of Exendin-9, a GLP-1 receptor antagonist. Mice were fasted for 6 hr and blood samples collected at time 0. PBS (a) or 0.12 mg/kg BW Exendin-9 (b) were i.p. injected. Five minutes later, WES (3000 mg/kg BW) (S, SE) or vehicle (V, VE) containing equal amount of glucose as in WES (258 mg/kg BW) were administered orally and blood samples collected at the time points indicated. (c) area under curve of (a) and (b). Data are mean ± SD with *n* = 4 for each group. *P < 0.05 and **P < 0.01 denote significant difference compared to vehicle-treated mice analyzed by Student's *t* test.

 To further assess the contribution of GLP-1 secretion to the acute hypoglycemic effects of WES, mice were orally administered a single dose of WES or vehicle containing equal amount of glucose as in WES. Serum glucose was monitored for 3 hr. At 30 and 60 min post-dosing, changes of serum glucose from time 0 were significantly lower in mice treated with WES, compared to those in the vehicle group (*P* < 0.05) (Figure 6(a)). This experiment was then repeated with prior intraperitoneal injection of Exendin-9, a GLP-1 receptor antagonist. As shown in Figure 6(b), pre-treatment with Exendin-9 significantly increased glycemic response in both groups and this response was not significantly different between the 2 groups. In Figure 6(c), changes in the glycemic response was further presented as the AUC of Figures 6(a) and 6(b). Without Exendin-9 pretreatment, the AUC of WES treated mice tended to be lower than that of the vehicle group (*P* = 0.07) (Figure 6(c)). In contrast, Exendin-9 pretreatment not only increased the AUC but also abolished the difference between the two groups (Figure 6(c)). These data further support a role of GLP-1 in the acute hypoglycemic activity of WES. 

## 4. Discussion

 Our hypothesis that BG might exert an incretin effect was supported by data obtained in this study. In vitro, the BG water extract (WE) and its fractions (WES, WEL and pf) stimulated GLP-1 secretion in the STC-1 murine enteroendocrine cells. In vivo, higher serum GLP-1, insulin and lower glucose were observed in mice orally administered with a single dose of WES for 30 min (Table 1), indicating that WES stimulated GLP-1 secretion in vivo as well. This acute hypoglycemic effect of WES was abolished by a prior ip injection of Exendin-9, a GLP-1 receptor antagonist (Figure 6). Moreover, a single oral dose of WE and WES significantly improved ipGTT (Figure 5). Mice fed a 5% BGP diet showed a significantly higher insulinogenic index in OGTT (Figure 1). All these lines of evidences supported a role of GLP-1 in the hypoglycemic effect of BG. Interestingly, the stimulation of GLP-1 secretion by WE and BGP-Bi can be inhibited by probenecid and U73122 and an aglycone form of a cucurbitane triterpenoids isolated from BGP, namely, 19-nor-cucurbita-5(10),6,8,22(E),24-pentaen-3*β*-ol (Compound 5) showed highest efficacy in stimulating GLP-1 in the STC-1 cells.

The STC-1 cell line used in this study was derived from an endocrine tumor that developed in the small intestine of a double transgenic mouse expressing the rat insulin promoters linked to SV40 large T antigen and to the polyomavirus small T antigen. This cell line expresses T2R family members, including mouse orthologs of rT2R1 (mT2R19), rT2R2 (mT2R23), rT2R3 (mT2R18), rT2R4 (mT2R7), rT2R6 (mT2R30), rT2R8 (mT2R2), rT2R9 (mT2R5), and rT2R12 (mT2R26) [18]. In addition, these cells can respond to stimulations of bitter tastants [18, 49]. It has been used to study the effects of nutrients and secretogogues on the GLP-1 secretion and the mechanism underlying [44, 50].

The observation that BGP water extract (WE), but not organic solvent extracts (EE & EAE), stimulated GLP-1 secretion is in accordance with numerous studies indicating the hypoglycemic activity of BG juice in vivo [51]. Both the small (WES) and large (WEL) molecular weight fractions were active in increasing GLP-1 secretion, indicating multiple components of WE are able to stimulate GLP-1 secretion. The stimulation of GLP-1 by both the insulin like-peptide-rich fraction from WE (Pf) and bitter compounds rich fraction, BGP-Bi, further supports this point. As sensors for nutrients and food components ingested in the intestine, the enteroendocrine cells are equipped with various receptors, including those for carbohydrates, lipids, protein as well as sweet and bitter taste substances, despite that not all sensing receptors have been characterized [11, 52]. 

The small molecular weight fraction (WES) of WE is mainly constituted by polar molecules, such as sugars, amino acids, small peptides, water soluble alkaloids and plant secondary metabolites. The large molecular weight fraction (WEL) of WE might contain proteins and water soluble dietary fibers. Sugars, amino acids, protein and soluble/fermentable fibers are known to stimulate GLP-1 secretion [11]. Our Pf fraction prepared as the acetone precipitate of acid soluble proteins from WE is presumably constitutents of WEL. This fraction also stimulated GLP-1 secretion but is less potent than the WEL. Intact proteins such as casein, codfish, egg, and wheat, but not soybean, ovomucoid and some peas have been shown to have pronounced effects on GLP-1 release in the STC-1 cells [50].

A large number of lipophilic plant secondary metabolites existed as glycoside form (saponin in a broad sense) and the solubility in water depends on the chain length and number of sugar moiety [53]. Numerous phytosteroidal glycosides and triterpenoid glycosides have been isolated and identified from BG. The aglycone part might be C27 phytosteroid, oleanolic acid type or cucurbitane type C30 triterpenoids. The cucurbitane type triterpenoid glycosdies have been named goyaglycosideds, momordicosides, karavilosides, Kuguaglycosides, and so forth. Indeed, many aglycone triterpenoid compounds isolated from BG were obtained by hydrolyzing the sugar moiety [34, 54]. 

The bitter taste receptor TAS2R16 mediates bitter taste in response to *β*-glucopyranosides [48]. The bitter principles in BG fruits, momordicosides K, L. [43] are *β*-glucopyranosides of cucurbitane triterpenoids. Our BGP-Bi fraction presumably contained quite a few compounds of this type. As expected, this fraction exhibited highest folds of maximal stimulation (Figure 2(c)) among the BG extracts/fractions tested in this study. Moreover, the stimulation was inhibited by probenecid, an inhibitor of human TAS2R16 (Figure 3(b)). Probenecid could inhibit human TAS2R16, TAS2R38 and TAS2R43, but not TAS2R31 and other non-TAS2R GPCR [45]. Our speculation that bitter taste receptors might be involved in the stimulating GLP-1 secretion need further investigations, since the orthologues of these human bitter taste receptors in mice and STC-1 cells and their specific agonists/antagonists remain unknown. 

The nutrient and tastants sensing in enetroendocrine cells are mainly mediated by G-protein coupled receptors [5, 6]. When tastant binds to taste receptors like bitter taste receptors, a conformational change occurs at the receptor level resulting in the activation of a series of signal transducers such as G protein *α*-gustducin, phospholipase C-beta 2 (PLC*β*2), inositol 1,4,5-trisphosphate receptor type 3 (IP3R3), and transient receptor potential (TRP) channels that eventually depolarize the cell through elevation of intracellular Ca^2+^ concentration [55]. Probenecid could inhibit TAS2R16, TAS2R38 and TAS2R43, but not TAS2R31 and other non-TAS2R GPCR [44]. Our results that probenecid and U73122 inhibited GLP-1 secretion induced by WE and BGP-bi (Figures 3(a) and 3(b)) suggested the involvement of PLC*β*2 signaling and bitter taste receptors in this effect. However, the inhibitions were not complete. It is not known whether the concentrations of these 2 inhibitors used in this study was high enough. The highest concentration of probenecid (1 mM) and U73122 (6 *μ*M) used in this study was the highest that would not affect cell viability within 24 hr. These were equal to or higher than the effective dose reported in the literature [45, 49]. Alternatively, it is also possible that other receptors/signaling pathways other than TAS2R16, TAS2R38 and TAS2R43 were involved in the stimulation of GLP-1 secretion by WE and BGP-bi.

The aglycone form of momordicoside L has been reported to have hypoglycemic activity (compound CH93 of [35]; Compound 5 of [54]). In addition, aglycone form of cucurbitane triterpenoid, such as Cucurbitacin B and E, also elicit bitter response through TAS2R10 and TAS2R14 [56]. We therefore tested the 6 aglycone triterpenoid previously isolated from BGP. Among these, Compound 5 and 6 stimulated high GLP-1 secretion. Compound 5 showed especially high efficacy (Figure 4(e)). This compound is characterized by an aromatic B ring. Compound 6 is characterized by an epoxy linkage between C19 and C5 and a hydroxyl group at side chain C23. 

Whether the increased GLP-1 secretion contributes to improved glucose homeostasis in vivo was further examined in high fat fed mice. As GLP-1 has a very short half-life in the circulation, we chose to use a single dose oral administration and examine the acute effect. Among WE, WES and WEL, WES showed best effect in improving ipGTT (Figure 5(a)). So we followed by testing the acute effect of WES on plasma GLP-1, insulin and glucose. Indeed, 30 min after oral administration of a dose of WES resulted in a significantly higher plasma GLP-1, insulin and lower glucose (Table 1). Moreover, this better plasma glucose control was abolished by pretreatment with a GLP-1 receptor antagonist Extendin-9. These data demonstrated that BGP extract simulated GLP-1 in vivo as well and contribute to a better glucose control.

In conclusion, this study provides evidences that BGP can exert an incretin effect which might contribute, at least in part, to a better glucose homeostasis control. To our knowledge, this is the first study demonstrating an incretin effect of BG.

## Figures and Tables

**Table 1:** Acute effects of WES on plasma glucose, insulin, and GLP-1 in mice^1^.

Group	Vehicle	WES
Plasma glucose (mg/dL)		
0 min	395.7 ± 24.1	377.6 ± 40.9
30 min	411.5 ± 22.4	282.6 ± 24.2***
30 min–0 min	015.8 ± 24.1	−95.0 ± 39.3^∗∗∗,##^
Plasma insulin (ng/mL)		
0 min	0.78 ± 0.29	0.66 ± 0.34
30 min	0.46 ± 0.09	0.80 ± 0.35**
30 min–0 min	−0.32 ± 0.32^#^	0.15 ± 0.26*
Plasma GLP-1 (pM)		
0 min	4.69 ± 1.96	4.60 ± 1.46
30 min	4.71 ± 1.86	8.18 ± 2.50***
30 min–0 min	0.02 ± 0.88	3.58 ± 1.38^∗∗∗,###^

^1^High-fat-diet-fed mice were fasted for 6 hr and blood samples collected at 0 min. Vehicle (430 mg/kg BW glucose, equal to glucose concentration in WES) or WES (5000 mg/kg BW) were orally administered and blood samples collected again after 30 minutes. Data were mean ± SD of two separate experiments with *n* = 3 for each group in each experiment. *P < 0.05, **P < 0.01 and ***P < 0.001 denote significant differences compared to vehicle-treated mice analyzed by Student's *t* test with RCBD to adjust for the differences between separate experiments. ^#^ *P* < 0.05, ^##^ *P* < 0.01, and ^###^ *P* < 0.001 denote significant differences between before and 30 min after treatment in the same group analyzed by paired *t* test.

## Abbreviations

BG: Bitter gourd
BGP: Bitter gourd powder
BGP-Bi: Bitter compounds rich fraction of BGP
DPP IV: Dipeptidylpeptidase IV
EA: Ethyl acetate
GLP-1: Glucagon like peptide-1
ipGTT: Intraperitoneal glucose tolerance test
OGTT: Oral glucose tolerance test
Pf: Insulin-like peptide-rich fraction of WE
PLC*β*2: Phospholipase C*β*2
PPAR: Peroxisome proliferator activated receptor
T2DM: Type 2 diabetes mellitus
WE: Water extract of BGP
WEL: Large molecule fraction of WE (>3 kD)
WES: Small molecule fraction of WE (<3 kD).
